# Massage Therapy for Fatigue Management in Breast Cancer Survivors: A Systematic Review and Descriptive Analysis of Randomized Controlled Trials

**DOI:** 10.1155/2021/9967574

**Published:** 2021-09-23

**Authors:** Tao Wang, Jianxia Zhai, Xian-Liang Liu, Li-Qun Yao, Jing-Yu (Benjamin) Tan

**Affiliations:** Charles Darwin University, College of Nursing and Midwifery, Brisbane Centre, 410 Ann Street, Brisbane, QLD 4000, Australia

## Abstract

**Background:**

Fatigue is one of the most common symptoms among breast cancer survivors. Although massage therapy has been commonly used for fatigue management, relevant evidence on the effectiveness of massage therapy for the reduction of fatigue in breast cancer survivors is still unclear.

**Objective:**

To identify the research evidence on the effectiveness and safety of massage therapy to manage fatigue in breast cancer survivors and summarize the characteristics of massage therapy protocols utilized for fatigue management in breast cancer survivors.

**Methods:**

Randomized controlled trials (RCTs) using massage therapy to manage cancer-related fatigue were searched in PubMed, Medline, Web of Science, Cochrane Library, Cumulative Index to Nursing and Allied Health Literature (CINAHL), ScienceDirect, PsycINFO, Wan Fang Data, and China National Knowledge Infrastructure (CNKI) from the inception of each database to March 2021. The Cochrane Back Review Group Risk of Bias Assessment Criteria was used to assess the methodological quality of the included studies. Descriptive analysis was applied for a summary and synthesis of the findings. The primary outcome was fatigue measured by any patient-reported questionnaires, and the secondary outcomes were quality of life and massage-therapy-related adverse events.

**Results:**

Ten RCTs were included. Massage therapy was found to have a positive effect on fatigue management compared with routine care/wait list control groups and sham massage. Despite these encouraging findings, the review concluded that most of the included studies exhibited an unsatisfactory experimental design, particularly, inadequate blinding and allocation concealment. The duration and frequency of the massage therapy interventions varied across the studies. Adverse events were reported in three included studies, with no study conducting causality analysis.

**Conclusion:**

This systematic review provides the latest research evidence to support massage therapy as an encouraging complementary and alternative medicine approach to managing fatigue in breast cancer survivors. More rigorously designed, large-scale, sham-controlled RCTs are needed to further conclude the specific therapeutic effectiveness and safety issues of massage therapy for fatigue management.

## 1. Introduction

Breast cancer is the world's most prevalent cancer and is a common risk factor that can reduce life expectancy, particularly among females [1]. Cancer-related fatigue (CRF) is one of the most debilitating symptoms experienced by breast cancer survivors, which is mainly caused by radiotherapy and chemotherapy [2]. The burden of unmanaged CRF can lead to reduction in quality of life (QoL) as it affects patients' physical function, mood, social interaction, and cognitive performance [3]. Cancer-related fatigue has a more significant negative effect on QoL than other cancer-related symptoms, such as pain, nausea, vomiting, and depression, and it can last for months or even years after cancer treatment [4].

Pharmaceutical agents that are commonly applied to manage CRF consist of antidepressants, steroids, cholinesterase inhibitors, donepezil, and stimulants [5]. However, evidence regarding their effectiveness and safety in breast cancer patients remains inconsistent and unclear [6]. Physical (such as high blood pressure and kidney/liver damage) and psychological (such as restlessness and anxiety) side effects and consequences pertaining to pharmaceutical interventions have impelled patients to turn to complementary and alternative medicine (CAM) as supplementary approaches to fatigue management [7]. Various CAM approaches, such as mindfulness-based interventions (e.g., yoga) [8], cognitive-behavioral therapy (CBT) [9], and physical exercise [10], have been used to manage fatigue as supplementary approaches. However, interventions such as yoga and physical exercise are energy-consuming, which may decrease patients' willingness to participate [11], particularly for those with significant fatigue symptoms. Other approaches such as CBT have a high cost and require extensive professional support, which can limit the space for long-term symptom management. Other nonpharmacological approaches that are less time- and energy-consuming are worthy of further exploration to facilitate better management of CRF in the long run.

Massage therapy has generally been considered a safe CAM approach to managing a wide range of health problems [12–17]. There are several types of commonly used massage therapy techniques in clinical practice, including Chinese massage, Japanese massage, Thai massage, Swedish massage, and reflexology. These types of massage involve handling muscles and stroking or rubbing the soft tissues of the human body [18], which can modulate body functions and cause relaxation [19–21]. Evidence has indicated that practicing massage therapy has a beneficial impact on increasing heart rate variability [22], improvement in mood disturbance [22–24], as well as QoL [25, 26] and reducing fatigue [22, 27] and physical discomfort [24]. Particularly, massage therapy has a great rate of acceptance and has been commonly applied in fatigue management among breast cancer survivors [28].

In the past few years, a growing body of small-scale clinical studies have been implemented to assess the effects of massage therapy on relieving fatigue in breast cancer patients, and some evidence has demonstrated that massage therapy decreased CRF [22, 23, 25, 29]. In addition, three systematic reviews/review protocols relating to massage therapy for cancer symptom management have been published [12, 14, 28]. However, the review by Finnegan-John et al. [12] focused on different types of CAM interventions for CRF management in patients with different cancer diagnoses, while the other two studies emphasized the effect of massage therapy on CRF relief [14, 28]. Pan et al. [14] generally addressed treatment-related side effects of breast cancer rather than focusing on fatigue management, and Wang et al. [28] study was a review protocol but without any available review findings, and it included all types of cancer diagnoses.

Since all the abovementioned systematic reviews/review protocols were published three years ago, and there was no evidence synthesis study that specifically focused on the use of massage therapy for fatigue relief among breast cancer survivors. It is, therefore, necessary to explore the latest research evidence on massage for fatigue management in breast cancer survivors by appraising more recent clinical research evidence from published randomized controlled trials (RCTs). This systematic review was conducted to explore the effectiveness and safety of massage therapy for fatigue management, as well as to summarize the characteristics of massage therapy protocols for managing fatigue in breast cancer survivors.

## 2. Methods

This systematic review was conducted and reported based on the PRISMA 2020 checklist for systematic reviews.

### 2.1. Data Sources and Search Strategies

This systematic review located studies from nine academic databases, including PubMed, Medline, Web of Science, Cochrane Library, Cumulative Index to Nursing and Allied Health Literature (CINAHL), ScienceDirect, PsycINFO, Wan Fang Data, and China National Knowledge Infrastructure (CNKI) from the inception of each database to March 2021. MeSH terms and keywords such as “massage,” “fatigue,” “lassitude,” “weariness,” “breast neoplasms,” and “breast cancer” were the primary search terms used for the electronic database search. Chinese MeSH terms and keywords, including 推拿/按摩, 疲劳/疲乏/癌因性疲乏/劳累, and 乳腺癌/乳腺肿瘤, were used for the CNKI and Wan Fang Data database search. The search strategy for PubMed is shown in Table 1. The reference lists of the retrieved literature were also reviewed to identify additional eligible studies.

### 2.2. Inclusion Criteria

Inclusion criteria were: (1) types of studies: randomized controlled trials (RCTs) conducted in any healthcare setting; (2) participants: adult breast cancer survivors, regardless of cancer stage, reporting fatigue; (3) intervention(s): massage therapy (any types of massage such as Chinese massage, Swedish massage, Japanese massage, Thai massage, reflexology, etc.); (4) control: wait list control, standard methods of treatment and/or care (usual care and/or standard medication), or other comparisons (placebo or sham control or other interventions) other than massage therapy; and (5) primary outcome: the symptom of fatigue measured by any patient-reported questionnaires, such as the Brief Fatigue Inventory (BFI), the Fatigue Severity Scale (FSS), and so on and secondary outcomes: QoL, safety outcomes, treatment satisfaction, and cost-effectiveness analysis. Chinese publications had to be indexed in the core journal list for methodological quality consideration.

### 2.3. Study Selection and Data Extraction

Two review authors (TW and JXZ) screened and identified eligible studies against the selection criteria independently using literature management software, EndNote X9. Eligible papers were included upon agreement of the same two reviewers. Any discrepancy in the selection and inclusion of a study was addressed by consulting with the third (JYT) and fourth (XLL) reviewers to determine eligibility. Data from the included studies were extracted adopting predefined forms, including: (1) characteristics of the included studies (e.g., authors, country, breast cancer stage, and study implementation); (2) description of massage therapy intervention protocols (e.g., massage modalities, procedure, intervention instructor, and timing, duration, and frequency); (3) methodological quality assessment (e.g., randomization, blinding, attrition, compliance, and dropouts); and (4) therapeutic effects of massage therapy (e.g., time points of assessment and fatigue-related outcomes). The third (JYT) and fourth (XLL) authors were consulted if a disagreement on data extraction emerged.

### 2.4. Quality Assessment of the Literature

The risk of bias and the methodological quality of each included study were assessed by two reviewers independently (TW and JXZ) using the Cochrane Back Review Group Risk of Bias Assessment Criteria [30]. The appraisal tool includes the following criteria: (1) “random sequence generation,” (2) “allocation concealment,” (3) “baseline assessment,” (4) “blinding – participants,” (5) “blinding – care provider,” (6) “blinding – outcome,” (7) “cointerventions,” (8) “compliance,” (9) “dropouts,” (10) “timing,” (11) “selective outcome reporting,” (12) “incomplete outcome data,” and (13) “other bias” (e.g., inclusion/exclusion criteria, sample size, reporting of adverse events, evaluation of therapeutic effects, and method of statistical analysis). Either “high risk of bias,” “unclear risk of bias,” or “low risk of bias” was adopted to rate each item. Further consultation with the third (JYT) and fourth (XLL) authors was conducted to settle any disagreements.

### 2.5. Data Analysis

The authors initially considered performing a meta-analysis using Review Manager. However, the notable heterogeneity in terms of the intervention protocols, comparisons, and outcome assessments made it an inappropriate method for carrying out a meta-analysis. Hence, narrative analysis was used to present the effects of massage therapy on fatigue among breast cancer survivors. In particular, narrative subgroup analysis was adopted for different comparisons, including massage therapy versus standard routine treatment/care or wait list control and massage therapy versus sham interventions (i.e., light touch and lay foot manipulation).

## 3. Results

**Figure 1:**
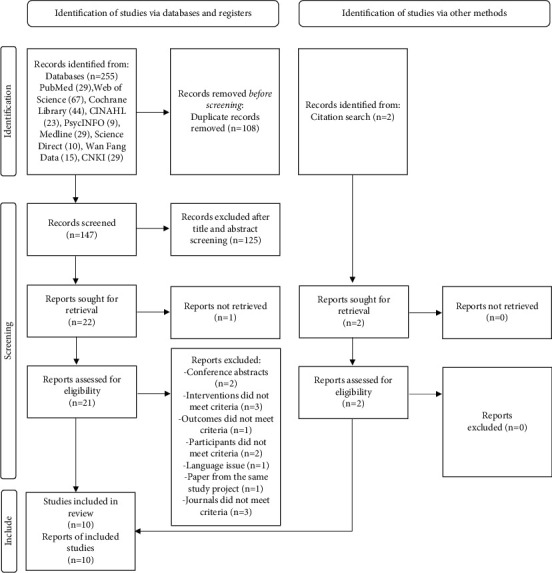
PRISMA flow diagram of the study selection. Adapted from: Page, M. J., McKenzie, J. E., Bossuyt, P. M., Boutron, I., Hoffmann, T. C., Mulrow, C. D. (2021). The PRISMA 2020 statement: An updated guideline for reporting systematic reviews. BMJ, 71, 372.

Of the 257 studies identified by searching the nine databases (*n* = 255) and other sources (manual retrieval, *n* = 2), 233 studies were removed after duplication checking and title and abstract screening. Twenty-four potentially eligible studies were located for further full-text assessment, of which 14 studies were excluded. Ten papers were retained for the systematic review, and the characteristics of the included studies were extracted (see Figure 1).

### 3.1. Characteristics of the Included Studies

This review included 10 studies, with 4 undertaken in the United States, 2 in Germany, and 1 each in China, Spain, Turkey, and Iran. In total, 1,040 randomized participants were involved in the current review, and 885 completed the studies (394 in intervention groups; 495 in control groups; completion rate = 79.59%). Only 2 studies [31, 32] had more than 100 study subjects. Seven of the included RCTs reported 2 arms to explore the effects of massage therapy by comparing massage therapy with standard routine care/wait list control. The remaining three RCTs [25, 26, 31] reported three arms, with two studies [25, 31] integrating a sham control group (light touch or lay foot manipulation).

Regarding the fatigue assessment tools, three studies [26, 27, 31] used the Brief Fatigue Inventory (BFI), and two studies [23, 24] adopted the Berlin Mood Questionnaire (BSF; fatigue subscale) for fatigue assessment. One each study employed the Fatigue Severity Scale (FSS) [33], the Chronic Fatigue Syndrome (CFS) Score [29], the MD Anderson Symptom Inventory [32], and the Profile of Mood States (POMS) Questionnaire (fatigue subscale) [22]. Noteworthily, Kinkead et al. [25] used the Multidimensional Fatigue Inventory (MFI) and the PROMIS Fatigue Short Form 7a (PROMIS) to evaluate fatigue. Mustian et al. [26] used two tools, including the BFI and daily fatigue diaries. Similarly, Listing et al. [24] adopted both the BSF (fatigue subscale) and the Giessen Inventory of Complaints (GBB; fatigue subscale) for outcome assessment. The characteristics of the reviewed studies are summarised in Table 2.

### 3.2. Massage Therapy Intervention Protocols

The characteristics of the massage protocols used in the included studies are shown in Table 3, including massage modalities, procedures, intervention instructors, timing of the interventions, and duration/frequency of the interventions. Four massage therapy modalities were identified in the review, of which reflexology therapy was adopted by four studies [27, 31–33], Swedish massage therapy was adopted by four studies [23–26], and Chinese massage therapy [29] and myofascial therapy [24] were adopted by one each study. Of the four studies that used reflexology therapy, one each was performed by a trained researcher [27], a trained caregiver [32], and certified reflexologists [31]. However, one study did not report the qualification of the intervention instructor [33]. Regarding the timing of the interventions, five studies carried out their intervention after primary treatment/chemotherapy/radiation therapy [22, 23, 25, 29, 33], while the remaining five conducted their intervention during chemotherapy or radiotherapy [24, 26, 27, 31, 32]. The duration of the massage interventions ranged from three weeks to three months. The frequency of massage therapy differed significantly across the included studies, ranging from 20 minutes twice/week to 45 minutes/week.

### 3.3. Quality Appraisal of the Included Studies

The quality appraisal results of the included studies are demonstrated in Table 4. Randomization was reported in all ten studies, with seven studies detailing their random sequence generation methods such as coin flips, computer-generated number sequences, and random number table [22–26, 29, 31]. Regarding allocation concealment, only three studies reported the use of sealed opaque envelopes [22, 25, 31]. For blinding, only one trial [31] reported the blinding of participants and care providers, and three trials [22, 25, 31] described the blinding of the outcome assessors. Six studies described the participants' dropout rates, and only one study reported dropouts exceeding 30% [23].

Of the ten studies, all the participants who completed the RCTs were analyzed, but only three [25, 31, 32] reported implementing intention-to-treat (ITT) analysis. For selective outcome reporting, all ten of the included studies were rated as low risk of bias. All ten studies conducted baseline assessments. Regarding “other bias,” inclusion/exclusion criteria of the participants, evaluation of therapeutic effects, and methods of data analysis were clearly elaborated in all the studies. However, only three studies [22, 25, 31] conducted a sample size calculation, and three studies described adverse events pertaining to practicing massage therapy.

### 3.4. Primary Outcome: Effects of Massage Therapy on Fatigue

The effects of massage therapy on fatigue management are outlined in Table 5. Narrative analysis was conducted to describe the effects of massage therapy on fatigue.

#### 3.4.1. Massage Therapy versus Standard Routine Treatment/Care or Wait List Control

Eight trials compared the effects of massage therapy with standard routine treatment/care or wait list control. Of these eight studies, four studies [22, 27, 29, 33] reported a statistically significant decrease in fatigue after the intervention compared with the routine care group (*p* < 0.01 or *p* < 0.05). Listing et al. [23] showed that, in comparing the two groups, the level of fatigue was lowered directly after the second treatment session but did not reach statistical significance (*p*=0.056). This decrease in fatigue was sustained over time and showed a statistically significant difference in the massage group compared with the control group at week 11 follow-up (*p*=0.01), and a similar result was reported by the study of Listing et al. [24]. Wyatt et al. [32] reported that there was a significant decrease in fatigue in the reflexology group compared with the control group at weeks 2 and 3 (*p* < 0.01), but no statistically significant differences between the intervention and control groups at week 4 (*p*=0.15). Mustian et al. [26] used the BFI as the primary fatigue measure and daily fatigue diaries as the secondary fatigue measure. The primary analysis revealed that the participants who received modified massage demonstrated a very small increase in CRF of 0.01 points (<1%) compared with the average increase in CRF of 0.25 points (13%) in the standard care group during weeks 1 to 3. However, the secondary analysis indicated an inconsistent finding, as the patients randomized to the modified massage group (0.59 points) had a greater increase in CRF than the standard care group (0.39 points) across all 3 weeks.

#### 3.4.2. Massage Therapy versus Sham Massage

Massage therapy was compared with sham massage in two trials, including light touch [25] and lay foot manipulation [31]. Of the two studies, Wyatt et al. [31] used the same BFI tool for fatigue assessment, while Kinkead et al. [25] applied the PROMIS. Wyatt et al. [31] reported that there was a statistically significant reduction in fatigue severity in the reflexology group compared with the lay foot manipulation group (*p*=0.02). The study conducted by Kinkead et al. [25]; which used the MFI tool for fatigue assessment, revealed large standardized treatment effect sizes, showing that Swedish massage therapy was statistically superior over light touch across time (effect size = 0.74; 95% CI = 0.10 to 1.38; *p* < 0.0001).

### 3.5. Secondary Outcome: Effects of Massage Therapy on QoL

Of the 10 studies, four RCTs measured and reported QoL as an outcome. Quality of life was assessed by 4 different questionnaires, including the Quality of Life Enjoyment and Satisfaction Questionnaire (Q-LES-Q) [25], the Quality of Life Index (QLI) [32], the Functional Assessment of Cancer Therapy – Breast (FACT-B) [31], and the Functional Assessment of Chronic Illness Therapy – Fatigue (FACIT-F) [26]. Regarding effectiveness, 2 studies reported group differences in measures of QoL as an outcome. Kinkead et al. [25] reported that the Q-LES-Q scores increased substantially for the intervention group compared with the light touch and wait list control group, and the results reached statistical significance over the 6-week trial period (*p*=0.0019). Mustian et al. [26] highlighted that the patients in the Swedish massage group reported less decline in health-related QoL than the standard care group, but no statistical significance was found (*p*=0.31, *p*=0.09, and *p*=0.64 for Week 1, Week 2, and Week 3, respectively). In contrast, Wyatt et al. [32] and Wyatt et al. [31] reported that no statistically significant differences were identified regarding QoL in the intervention group compared with the sham control/standard care group.

### 3.6. Secondary Outcome: Adverse Events

Of the 10 trials, 3 [23, 25, 31] mentioned adverse events in the Results section, of which 1 study reported no adverse effects [31]. Kinkead et al. [25] set the adverse events as safety outcomes, and they were monitored at each therapy session, with bruising at the venipuncture site observed in 12/39 participants and discomfort caused by hypertension from lying on the table experienced by 2/39 subjects. Listing et al. [23] reported that 1 participant had higher back pain and another participant experienced an increase in blood pressure, but those adverse events were resolved in a later massage session. None of the 10 included studies indicated causality analysis protocols for monitoring massage therapy-related adverse events.

### 3.7. Satisfaction with Treatment

None of the reviewed studies reported findings on treatment satisfaction.

### 3.8. Cost-Effectiveness

Cost-effectiveness analysis was not conducted by any of the reviewed trials.

## 4. Discussion

Considering the relatively small number of studies analyzed in this review and the unsatisfactory methodological quality of some included studies, the current evidence remains inconclusive but does support the promising role of massage therapy in alleviating fatigue in breast cancer survivors. Mustian et al. [6] stated that the effectiveness of massage therapy on fatigue was related to the stage of cancer, preliminary treatment status, experimental treatment delivery method, type of control condition, employment of intention-to-treat analysis, and fatigue measurement tools. Therefore, the results should be interpreted prudently.

### 4.1. Summary of Primary Outcome

Although meta-analysis was not conducted, the findings via descriptive analysis demonstrated that massage therapy had a positive effect on fatigue management in breast cancer survivors compared with those who received standard routine care/wait list control and sham massage. Consistent with the systematic review conducted by Pan et al. [14], the current review suggested that there was a greater alleviation of fatigue symptoms among the breast cancer survivors who received massage therapy compared with the control group. Regarding the effect of sham massage (i.e., light touch and lay foot manipulation), the current review revealed that both sham massage modalities demonstrated superiority over standard care/wait list control. In particular, there was a significant reduction in fatigue after applying lay foot manipulation, suggesting that this modality may be a beneficial addition to adjunctive care for survivors with breast cancer [31].

### 4.2. Summary of Secondary Outcomes

Regarding QoL, inconsistent findings were revealed in this review, which was in accordance with the review by Pan et al. [14], suggesting that massage therapy can potentially ameliorate QoL among cancer patients. Regarding adverse effects, one argument for the application of massage therapy for breast cancer symptom management is that it has few adverse reactions [34]. However, none of the included trials provided information regarding precautions of any potential adverse reactions associated with massage therapy. There was also no information about causality assessments between the massage therapy interventions and the adverse events that occurred in the reviewed studies. Therefore, evidence regarding the safety of massage therapy remains unclear. Adverse effects should be noted in future studies.

This review identified that none of the reviewed trials evaluated treatment satisfaction or conducted the cost-effective analysis. In the systematic review conducted by Barbosa et al. [35], it was suggested that there is a positive statistical association between treatment satisfaction and compliance, adherence, and lower treatment burden for different spectrums of diseases in clinical trials. Inadequate compliance in RCTs can lead to poor quality studies and reduce therapeutic outcomes [36]. None of the reviewed studies adopted validated outcome measures to explore cost-effectiveness relationships related to performing massage therapy. A recent review has indicated that cost-effectiveness analysis is the standard approach in health economics [37]. The benefits of health economics are uncertain without an assessment of the cost-effectiveness of massage therapy in RCTs [37, 38].

### 4.3. Intervention Protocols

Although the majority of the included studies described the massage therapy intervention protocols, none of the studies elaborated whether the intervention protocol was developed based on current best available evidence and the guidance of frameworks, such as the Medical Research Council (MRC) framework for complex interventions. In addition, there were variations in the interventions' modalities, the pluralism of massage therapy, the expertise of the intervention instructors, and the descriptions of frequency and duration in the included studies.

Of the four intervention modalities, reflexology therapy and Swedish massage therapy were the most frequently utilized among the included trials. Nevertheless, considering the relatively limited number of included studies, it was challenging to determine the most suitable modality of massage therapy for fatigue management. In addition, the massage therapy was performed by various instructors, including trained researchers, caregivers, nurses, massage therapists, and so on, which could have had an impact on the effect and safety of massage therapy practice. Furthermore, variations in massage therapy duration and frequency were also observed, which indicated that standard massage therapy practice with evidence-based intervention components is scant. From the descriptive analysis, it was feasible to perform massage therapy 30 to 45 minutes/session, one to two times per week. No consistent massage therapy protocol has been observed with sufficient sample sizes to date.

### 4.4. Quality of the Evidence

This systematic review appraised the methodological quality of the included studies using the Cochrane Back Review Group Risk of Bias Assessment Criteria. Of the ten included studies, only one trial blinded the participants and care providers, and three trials blinded the outcome assessors. The other six studies did not report blinding information, which implies a potential detection bias [39]. Allocation concealment was also reported in only three studies. Clinical studies without adequate allocation concealment and blinding design are likely to introduce a selection bias that can produce exaggerated intervention effects, which can impact the reliability of the trials' findings [40]. Similarly, ITT analysis was described in only three trials, which may have been subject to an attrition bias [41].

### 4.5. Study Limitations

Although all the included trials suggested that massage therapy generates beneficial effects, the trials reviewed had variable quality, which may have prohibited drawing any firm and consistent conclusions. Besides the flaws in methodological quality, the primary limitation of the reviewed studies was significant clinical heterogeneity, including insufficient sample sizes, different types of massage therapy, study comparisons, intervention duration, and no or short follow-up periods. Regarding the limitations of this systematic review, there was the possibility of language bias given that only Chinese and English literature were searched and included. Although the electronic searches were extensive and considered grey literature as well, the review was not able to guarantee that all pertinent studies were located. It is possible that studies with negative findings were not published and therefore could not be identified. Hence, publication and reporting biases may exist.

### 4.6. Implications for Further Research and Practice

The review findings provided preliminary research evidence to support the use of massage therapy as a promising approach to alleviating fatigue in breast cancer survivors in clinical practice. Given the variations in the intervention protocols in the analyzed studies, in future research, developing evidence-based massage therapy protocols with an appropriate modality and most favorable duration and frequency is warranted. More well-designed multicentered RCTs with appropriate sham massage therapy designs and adequate sample sizes are needed to provide more robust evidence on massage therapy for fatigue management in breast cancer survivors. Moreover, this review highlighted some methodological issues that can be further enhanced in future studies. First, the protocols of the massage therapy, including massage modality, intervention duration and frequency, and qualifications of the instructors, should be fully described with justifications. Second, blinding data collectors and outcome assessors should be considered to reduce the effects of patients' expectations on the measured outcomes. Moreover, interventions with follow-up periods should be designed to monitor the long-term effects of massage therapy. In addition, adverse events and the causality between massage therapy and adverse events should be fully measured and reported. Furthermore, some valid objective measurements such as physiological sensors should be considered in future research to provide a comprehensive assessment of fatigue. Treatment satisfaction with and cost-effectiveness of massage therapy should also be evaluated in future studies to identify the acceptability and feasibility of the wide use of massage therapy in clinical practice.

## 5. Conclusion

This study identified a potentially favorable role of massage therapy in reducing cancer-related fatigue in breast cancer survivors. However, evidence on the definite effects of massage therapy for fatigue management in breast cancer survivors is inconclusive due to some limitations in quantity and quality identified in the included studies. More rigorously designed, sham-massage RCTs with large sample sizes are warranted to minimize study bias and yield high-quality and robust evidence.

## Figures and Tables

**Table 1:** A representative search strategy (PubMed).

ID	Search strategy
#1	“massage”[MeSH Terms]
#2	(((“massage”[Title/Abstract]) OR (“massage therapy”[Title/Abstract])) OR (“massage therapies”[Title/Abstract])) OR (“tuina”[Title/Abstract])
#3	#1 OR #2
#4	(“fatigue”[MeSH Terms]) OR (lassitude[MeSH Terms])
#5	((((((“fatigue”[Title/Abstract]) OR (lassitude[Title/Abstract])) OR (tired*∗*[Title/Abstract])) OR (“weary”[Title/Abstract])) OR (“weariness”[Title/Abstract])) OR (exhaust*∗*[Title/Abstract])) OR (“lacklustre”[Title/Abstract])
#6	#4 OR #5
#7	breast neoplasms[MeSH Terms]
#8	(((((((“breast neoplasms”[Title/Abstract]) OR (“breast neoplasm”[Title/Abstract])) OR (“breast tumors”[Title/Abstract])) OR (“breast tumor”[Title/Abstract])) OR (“breast cancer”[Title/Abstract])) OR (“breast carcinoma”[Title/Abstract])) OR (“mammary tumor”[Title/Abstract])) OR (“mammary cancers”[Title/Abstract])
#9	#7 OR #8
#10	#3 AND #6 AND #9

**Table 2:** Characteristics of the included studies.

Study	Country	Study design	Cancer stage	Sample size and age	Intervention	Control	Outcomes
S1: [33]	Iran	Double-blind RCT	Stage I	Randomized: 60 Completed: 57 Intervention G: 27/30, age (yr) = 47.85 ± 8.39 Control G: 30/30, age (yr) = 50.86 ± 6.5	Reflexology therapy	Routine treatment and care	Fatigue: Fatigue Severity Scale (FSS)
S2: [29]	China	RCT	Stages I–IV	Randomized: 98 Completed: 98 Intervention G: 49/98, age (yr) = 50.76 ± 10.25 Control G: 49/98, age (yr) = 50.31 ± 10.79	Chinese massage therapy	Routine treatment and care	Fatigue: Chronic Fatigue Syndrome (CFS)
S3: [25]	USA	Single-blind RCT, three groups	Stages 0–III	Randomized: 66 Completed: 56 Group A: 20/22, age (yr) = 54.5 ± 12.4 Group B: 19/22, age (yr) = 55.6 ± 9.0 Group C: 17/22, age (yr) = 51.8 ± 9.6	Group A Swedish massage therapy	Group B: light touch (LT) Group C: wait list control (WLC)	Fatigue: Multidimensional Fatigue Inventory (MFI) + PROMIS Fatigue Short Form 7a (PROMIS) QoL: Quality of Life Enjoyment and Satisfaction Questionnaire (Q-LES-Q) Safety: adverse events
S4: [27]	Turkey	RCT	Stages I–III	Randomized: 60 Completed: 60 Intervention G: 30/30, age (yr) = 50.93 ± 11.27 Control G: 30/30, age (yr) = 51.06 ± 10.97	Reflexology therapy	Routine treatment and care	Fatigue: Brief Fatigue Inventory (BFI)
S5: [32]	USA	RCT	Stages III and IV	Randomized: 256 (patient-caregiver dyads) Completed: 207 Intervention G: 92/128, age (yr) = 58 (mean age) Control G: 99/128, age (yr) = 55 (mean age)	Reflexology therapy	Routine treatment and care	Fatigue: MD Anderson Symptom Inventory QoL: Quality of Life Index (QLI)
S6: [22]	Spain	Single-blind, placebo-controlled crossover design	Stage I–IIIa	Randomized: 20 (crossover design) Completed: 20 Intervention G: 20/20, age (yr) = 49.1 ± 7.8 Control G: 20/20, age (yr) = 49.1 ± 7.8	Myofascial massage	Routine treatment and care	Fatigue: Profile of Mood States (POMS) Questionnaire (fatigue subscale)
S7: [31]	USA	Longitudinal, randomized clinical trial	Stages III and IV, or stages I and II with recurrence or metastasis	Randomized: 286 Completed: 243 Group A: 83/95, age (yr) = 55.3 ± 9.4 Group B: 83/95, age (yr) = 54.8 ± 11.2 Group C: 77/96, age (yr) = 57.3 ± 11.8	Group A: reflexology therapy	Group B: lay foot manipulation (LFM) – light touch Group C: routine treatment and care	Fatigue: Brief Fatigue Inventory (BFI) QoL: Functional Assessment of Cancer Therapy – Breast (FACT-B) Safety: adverse events
S8: [26]	USA	RCT	Any stage	Randomized: 45 Completed: 43 Group A: 15/15 Group B: 13/13 Group C: 15/15 age (yr) = 25.8	Group A: modified Swedish massage therapy	Group B: polarity therapy Group C: routine treatment and care	Fatigue: Brief Fatigue Inventory (BFI) Daily fatigue diaries (a 0–10 scale) QoL: Functional Assessment of Chronic Illness Therapy-Fatigue (FACIT-F)
S9 [23]	Germany	RCT	Any stage	Randomized: 34 Completed: 29 Intervention G: 16/17, age (yr) = 59.5 ± 12.1 Control G: 13/17, age (yr) = 59.9 ± 11.5	Swedish massage therapy	Routine treatment and care	Fatigue: Berlin Mood Questionnaire (BSF; fatigue subscale)
S10: [24]	Germany	RCT	Without distant metastases	Randomized: 115 Completed: 72 Intervention G: 44/58, age (yr) = 57.6 ± 10.8 Control G: 28/57, age (yr) = 61.4 ± 10.9	Swedish massage therapy	Routine treatment and care	Fatigue: Berlin Mood Questionnaire (BSF; fatigue subscale) Giessen Inventory of Complaints (GBB; fatigue subscale)

*Note.* QoL = quality of life.

**Table 3:** Description of massage therapy interventions.

Study	Massage modality	Massage procedure	Intervention instructor	Timing of intervention	Intervention duration	Frequency	Follow-up
S1: [33]	Reflexology therapy	Pressing the major reflexive points of the soles with the thumb and index finger in a worm-like movement	NR	After chemotherapy	4 weeks	Twice per week, 20 min per session	No
S2: [29]	Chinese massage therapy	(1) Massaging the patient's *Zusanli*, *Yongquan*, *Neiguan*, *Guanyuan*, *Baihui*, *Shenmen*, and temple points (2) Massaging and beating of acupuncture point on the affected side of the patient's affected limb that had limited mobility	Specialist nurses	After surgery	3 months	Twice per week, around 30 min per session	No
S3: [25]	Swedish massage therapy	SMT techniques using effleurage kneading of underlying muscles and tapotement (1) Patient takes a prone position while the therapist performs massage from the shoulders to the feet (2) Patient turns to a supine position and the massage therapist continues with the intervention from the feet to the shoulders, and then the head	Licensed massage therapists	After primary treatment	6 weeks	Weekly, 45 min per session	No
S4: [27]	Reflexology therapy	(1) Performing primary relaxation techniques (effleurage, shaking, rotation, and stretching) on both feet (2) Performing reflexology techniques on all organ systems	Trained researcher	During chemotherapy	Three sessions (one in each chemotherapy cycle, 21 days)	Around 30–40 min each session	No
S5: [32]	Reflexology therapy	Performing nine reflexes on the foot with thumb-walking pressure	Trained caregivers	During chemotherapy, targeted, or hormonal therapy	4 weeks	Weekly, 30 min per session	11 weeks
S6: [22]	Myofascial massage	Performing pressure, stroke, ear pull, and frontalis bone spread skills on the neck-shoulder area with the Barnes approach	Physical therapist	After coadjuvant treatment except hormone therapy	NR	Two occasions separated by a 2-week interval, 40 min per session	No
S7: [31]	Reflexology therapy	Stimulating the nine essential reflexes specifically relating to breast cancer using reflexology (deep thumb-walking pressure)	Certified reflexologists	During chemotherapy	4 weeks	Weekly, 30 min per session	No
S8: [26]	Modified Swedish massage therapy	Applying strokes, including light moving touch, compression, and static holds technique	Licensed massage therapists	During radiation therapy	3 weeks	Weekly, 30 min per session	No
S9: [23]	Swedish massage therapy	Applying stroking, friction, kneading skills to the patients in a prone position. Muscles for massage: compendiously neck muscles, autochthonal back muscles, scapulae, trapezii, latissimi dorsi, supraspinati, teres majores, pectorales majores, and so on	Licensed, trained female massage therapist	After primary treatment	5 weeks	Twice a week, 30 min per session	11 weeks
S10: [24]	Swedish massage therapy	Same as [24]	Licensed, trained female massage therapist	After chemotherapy and/or radiation therapy	5 weeks	Twice a week, 30 min per session	11 weeks

*Note.* NR = not reported.

**Table 4:** Methodological quality appraisal of the included studies.

	Criteria	S1: [33]	S2: [29]	S3: [25]	S4: [27]	S5: [32]	S6: [22]	S7: [31]	S8: [26]	S9: [23]	S10: [24]
1	Random sequence generation	⨯	✓	✓	⨯	⨯	✓	✓	✓	✓	✓
2	Allocation concealment	?	?	✓	?	?	✓	✓	?	⨯	⨯
3	Baseline assessment	✓	✓	✓	✓	✓	✓	✓	✓	✓	✓
4	Blinding – participants	?	?	⨯	?	?	⨯	✓	⨯	?	?
5	Blinding – care provider	?	?	⨯	?	?	⨯	✓	⨯	?	?
6	Blinding – outcome	?	?	✓	?	?	✓	✓	⨯	?	?
7	Cointerventions	✓	✓	✓	✓	✓	✓	✓	⨯	✓	✓
8	Compliance	✓	✓	✓	?	✓	?	✓	⨯	✓	✓
9	Dropouts	⨯	✓	✓	?	✓	?	✓	✓	✓	⨯
10	Timing	✓	✓	✓	✓	✓	✓	✓	✓	✓	✓
11	Selective outcome reporting	✓	✓	✓	✓	✓	✓	✓	✓	✓	✓
12	Incomplete outcome data	⨯	⨯	✓	?	✓	?	✓	⨯	⨯	⨯
**13**	**Other bias**										
	Sample size calculation	?	?	✓	⨯	?	✓	✓	?	⨯	⨯
	Inclusion criteria	✓	✓	✓	✓	✓	✓	✓	✓	✓	✓
	Exclusion criteria	✓	✓	✓	✓	✓	✓	✓	✓	✓	✓
	Evaluation of treatment effects	✓	✓	✓	✓	✓	✓	✓	✓	✓	✓
	Adverse events reporting	⨯	⨯	✓	?	?	?	✓	?	✓	?
	Data analysis methods	✓	✓	✓	✓	✓	✓	✓	✓	✓	✓

*Note.* ⨯: high risk; ✓: low risk; and ?: unclear.

**Table 5:** Effects of the massage therapy on CRF.

Study	Intervention (mean ± SE)	Control (mean ± SE)	Assessment time points	Fatigue outcome measures	Description of the effects
S1: [33]	20.66 ± 4.54	40.36 ± 9.58	Postintervention (4 weeks)	Fatigue Severity Scale (FSS)	Significant difference was identified between the intervention and control groups (*p* ≤ 0.01)
S2: [29]	2.63 ± 1.71	3.61 ± 2.16	Postintervention (3 months)	Chronic Fatigue Syndrome (CFS)	Statistical differences were identified between the intervention and control groups (*p* < 0.05)
S3: [25]	NR	NR	Postintervention (6 weeks) at visits 3 and 6 weeks	Multidimensional Fatigue Inventory (MFI) and Fatigue Short Form 7a (PROMIS)	**Mixed model repeated measures analysis:** the Swedish massage group showed statistically better outcomes over the light touch and the wait list control groups, as well as for superiority of the light touch over the wait list control over time (*p* < 0.0001) **PROMIS analysis:** significant improvement of fatigue for the Swedish massage group and the light touch group over 6 weeks, while remaining the same for the wait list control group
S4: [27]	1.20 ± 1.44	2.33 ± 1.65	Postinterventions (every chemotherapy cycle)	Fatigue: Brief Fatigue Inventory (BFI)	Differences were observed between the intervention and control groups in the onset and first, second, and third measurements (*p* < 0.05)
S5: [32]	**W2:** 3.36 ± 0.24 **W3:** 3.75 ± 0.24 **W4:** 3.57 ± 0.24	**W2:** 4.95 ± 0.24 **W3:** 4.63 ± 0.24 **W4:** 4.23 ± 0.24	Postintervention(4 weeks)	MD Anderson Symptom Inventory	Significant reduction in fatigue severity was identified in the intervention group compared with the control group beginning at weeks 2 and 3 (*p* < 0.01) No statistically significant differences between the two groups were identified for the severity of fatigue at week 4 (*p*=0.15)
S6: [22]	41.3 ± 4.9	43.4 ± 7.0	Postintervention	Fatigue: Profile of Mood States (POMS) Questionnaire (fatigue subscale)	Significant reduction in disturbance of mood and fatigue were observed after manual therapy (*p* < 0.001)
S7: [31]	5.9 ± 2.8	LFM G: 5.4 ± 3 Control G: 6 ± 2.8	Postintervention (4 weeks)	Brief Fatigue Inventory (BFI)	Significant reductions in fatigue severity in the intervention group was observed compared with the control group (*p* < 0.01) and the LFM group (*p*=0.02)
S8: [26]	**BFI:** 3.0 ± 2.2 **Daily fatigue diaries:** 4.5 ± 2.1	**BFI:** Modified Massage G: 3.6 ± 2.8 Control G: 2.5 ± 1.5 **Daily fatigue diaries** Polarity G: 4.5 ± 2.8 Control G: 3.2 ± 1.8	Postintervention (4 weeks)	Brief Fatigue Inventory (BFI) Daily fatigue diaries	**BFI analysis:** participants who received modified massage demonstrated a smaller increase in fatigue assessment of 0.01 points (<1%) compared with an average increase in fatigue assessment of 0.25 points (13%) in the standard care group during weeks 1 to 3 **Daily fatigue diaries analysis:** the patients randomized to modified massage had a greater increase (0.59 point) in CRF than the standard care group (0.39 point) across all 3 weeks
S9 [23]	**T2:** 18.2 ± 14.8 **T3:** 18.9 ± 14.8	**T2:** 27.9 ± 17.2 **T3:** 33.8 ± 16.4	Postintervention (5 weeks)	Fatigue Severity Scale (FSS)	Improvement of tiredness nearly reached statistical significance immediately after intervention (T2; *p*=0.056). A better improvement of tiredness was identified at T3. Statistically significant difference was identified between groups at follow-up (*p*=0.01).
S10: [24]	NR	NR	Postintervention (5 weeks)	Giessen Inventory of Complaints (GBB; fatigue subscale)	Fatigue was improved at the end of the treatment (*p*=0.06). Statistically significant difference was identified in the intervention group compared with the control group at week 11 (*p*=0.048).

*Note.* NR = not reported.

## Data Availability

Data that were used for analysis in this review were all extracted from the original studies. All data relevant to the study are included in the article.
